# Emotional Violence is Associated with Increased HIV Risk Behavior Among South African Adolescent Girls and Young Women in the HPTN 068 Cohort

**DOI:** 10.1007/s10461-021-03535-y

**Published:** 2021-11-20

**Authors:** Anna M. Leddy, Amanda Selin, Sheri A. Lippman, Linda J. Kimaru, Rhian Twine, Xavier Gómez-Olivé, Kathleen Kahn, Audrey Pettifor

**Affiliations:** 1 Department of Medicine, University of California, San Francisco, 550 16th St., 3rd floor, San Francisco, CA 94158 USA; 2 Stanford University School of Medicine, Stanford, CA USA; 3 MRC/Wits Rural Public Health and Health Transitions Research Unit (Agincourt), Faculty Health Sciences, School of Public Health, University of the Witwatersrand, Johannesburg, South Africa; 4 Department of Epidemiology, Gillings School of Public Health, University of North Carolina at Chapel Hill, Chapel Hill, NC USA; 5 Department of Public Health and Clinical Medicine, Umeå Centre for Global Health Research, Umeå University, Umeå, Sweden

**Keywords:** Emotional violence, Intimate partner violence, Adolescent girls and young women, HIV risk behaviors, Sub-Saharan Africa

## Abstract

Limited research has explored how emotional intimate partner violence (IPV) shapes HIV risk behaviors. Using cross-sectional data from the HPTN 068 post-trial visit (N = 1942), we assessed the association between emotional IPV and its sub-domains (verbal abuse and threats) with condomless sex, transactional sex, and frequent alcohol use among young women in South Africa. In adjusted multivariable logistic regression models, any emotional IPV and verbal IPV were associated with increased odds of condomless sex (aOR: 1.47; 95% CI: 1.15, 1.87; and aOR: 1.48; 95% CI: 1.15, 1.89), transactional sex (aOR: 2.32; 95% CI: 1.74, 3.08; and aOR: 2.02; 95% CI: 1.51, 2.71) and alcohol use (aOR: 1.88; 95% CI: 1.39, 2.53; and aOR: 1.87; 95% CI: 1.37, 2.55). Threats were associated with transactional sex (aOR: 3.67; 95% CI: 2.62, 5.14). Future research should examine this relationship over-time and HIV prevention programs should consider and address emotional IPV.

## Introduction

Globally, adolescent girls and young women (AGYW) ages 15–24 years in sub-Saharan Africa are disproportionately affected by HIV and intimate partner violence (IPV) [1, 2], defined as physical, sexual and/or emotional violence perpetrated by a current or former partner [3]. AGYW in sub-Saharan Africa account for 25% of all new HIV infections globally [1], with the highest burden of new infections occurring among AGYW in South Africa [4]. The HIV prevalence among young South African women ages 15–19 years is 5.9%, rising to 16.7% among young women ages 20–24 years, indicating elevated HIV risk during this transitional period [5]. Sub-Saharan Africa is also the region with the highest prevalence of lifetime physical and sexual IPV among AGYW (34.4%) in the world [2]. Similarly, the prevalence of IPV increases substantially during the period of transition from adolescence to young adulthood. A recent study from rural South Africa found that the prevalence of lifetime IPV rose from 17.7 to 32.1% among AGYW ages 15–16 and 17–20 years, respectively [6].

IPV is a well-established determinant of HIV-acquisition risk [7–10] and has been identified as a factor driving the extremely high rates of HIV among AGYW in South Africa [7, 11, 12]. IPV can increase women’s risk for HIV directly through forced sex and indirectly through pathways including poor mental health, substance use, and limited agency to negotiate condom use [13, 14]. However, the vast majority of research examining the relationship between IPV and HIV acquisition risk has focused on physical and sexual IPV [15]. A small body of research has demonstrated an association between emotional IPV and HIV risk among women [8, 16, 17]. Emotional IPV includes the use of verbal and non-verbal communication with the goal of exerting control over a partner and/or harming them mentally or emotionally [18]. A cross-sectional study of data from demographic health surveys from 10 countries in sub-Saharan Africa found that emotional violence was associated with increased odds of HIV prevalence among ever-married and currently married women [8]. Another cross-sectional study among young South African women found that past year emotional IPV prevalence was extremely high (78.1%) and associated with reduced odds of condom use at last sex [16]. Much of this work has focused on adult women, with very few studies examining this relationship among AGYW ages 15–24 years. Such research is needed given the disproportionate burden of IPV and HIV among this population, as well as their unique developmental stage which may differentially influence their response to experiences of emotional IPV, with potentially negative implications for HIV prevention. To address this knowledge gap, we examined the relationship between emotional IPV and HIV risk behaviors among AGYW participating in the HPTN 068 trial in rural South Africa [19].

## Methods

### Study Setting and Procedures

HPTN 068 was a phase 3 randomized controlled trial of cash transfers conditional on school attendance among AGYW in the Bushbuckridge sub-district in Mpumalanga province South Africa [19]. This study area is the site of the Agincourt Health and Socio-Demographic Surveillance System (HDSS) where the Medical Research Council and University of the Witwatersrand Rural Public Health and Health Transitions Research Unit maintain a research platform including an annual population census, updating household rosters and collecting information on demographic events [20]. AGYW ages 13–20 were eligible to participate in the HPTN 068 trial if they were currently residing in the Agincourt HDSS study area and were enrolled in grades 8–11 at local government schools in 2011. Consenting participants were randomized 1:1 to receive a conditional cash transfer or control condition. All participants completed an Audio Computer-Assisted Self-Interview (ACASI) and HIV counselling and testing at baseline and up to three annual follow-up visits during the trial and an additional post-trial visit. We added a lifetime emotional IPV measure to the post-trial visit survey, which was conducted between 2015 and 2017, approximately 1–2 years after the final main study visit when participants were between 17 and 26 years of age. Parenting and economic data was also collected from the heads of the households in which AGYW resided. Details of the HPTN 068 trial have been published previously [19].

### Measures

#### Outcomes

Outcome variables were measured at the post-trial visit and included self-reported condomless sex, transactional sex, and frequent alcohol use. To assess condomless sex, participants were asked how many times they had vaginal sex in the past 3 months and were then asked of those times, how many times they used a condom. Similar questions were asked for anal sex in the past 3 months. Participants were classified as having had any condomless sex if the number of times they had vaginal or anal sex in the past 3 months was greater than the number of times they reported using a condom during vaginal or anal sex in the past 3 months.

Transactional sex was measured by showing participants a list of eight items including food, money, school fees, and other goods, and asking them to indicate (yes/no) if they had sex with a man in exchange for each item since the last study visit. A binary variable was created to capture any or no transactional sex since the last study visit (final main study visit), which occurred approximately 1–2 years prior.

Frequent alcohol use was assessed by a question asking participants to indicate how often they drink alcohol. Response options ranged from never (0) to daily (6). Frequent alcohol use was defined as drinking alcohol once a month or more vs. drinking alcohol less than once a month or never, based on the distribution of the data.

#### Exposures

The exposure was lifetime emotional violence from an intimate partner, measured by the previously validated 4-item World Health Organization (WHO) scale [21]. The WHO emotional violence measure is composed of two domains: verbal IPV and threats from a partner. Verbal IPV was assessed by the two items “Has your partner ever insulted you or made you feel bad about yourself?” and “Has a partner ever belittled or humiliated you in front of other people?” Threats were assessed by the two items “Has a partner ever done things to scare you or intimidate you on purpose, for example, by the way he looked at you, by yelling or smashing things?” and “Has a partner ever hurt people you care about as a way of hurting you, or damaged things of importance to you?” Response options to all items included: never, once, a few times, and many times. Responses were aggregated for overall emotional IPV and for each domain and dichotomized to reflect any vs. no violence.

#### Covariates

Covariates were selected based on prior research on factors associated with IPV and HIV risk behaviors, and included age at post-trial visit, current educational status (in school/graduated high school vs not attending school/dropped out), HPTN 068 intervention arm (vs. control), lifetime experiences of physical IPV and household assets. Lifetime experiences of physical IPV was assessed using the previously validated 6-item WHO scale [21]. The scale measures six types of physical violence perpetrated by a partner, ranging from being slapped to the use of a weapon. HPTN 068 participants were asked about lifetime experiences of physical violence at enrollment and experiences of physical violence in the past 12 months at the three follow-up visits and the post-trial visit. A binary variable was created for lifetime experiences of physical violence at the post-trial visit which captured AGYW who reported lifetime physical violence at enrollment or any recent physical violence at the follow-up visits prior to the post-trial visit. Household assets (the total number of durable goods from a list of 27 items each household owned) was measured in the household-survey and used to assess the socioeconomic status (SES) of AGYW and their families.

### Analysis

We conducted a cross-sectional secondary data analysis to examine the relationship between lifetime emotional violence and recent HIV risk behaviors using the HPTN 068 post-trial visit survey data- the only visit where emotional violence data were collected. A total of 1942 AGYW were included in this analysis. Descriptive analyses examined lifetime experiences of emotional IPV, recent transactional sex, frequent alcohol use, condomless sex in the past 3 months, and other covariates of interest. Key sociodemographic characteristics were compared between those individuals reporting lifetime experiences of emotional violence (n = 487) and those who did not (n = 1455) to examine potential differences using Pearson’s χ^2^ tests for binary or categorical variables and t-tests for continuous variables.

Separate bivariate logistic regression models assessed the association between the summary and sub-domain measures of lifetime emotional violence and covariates with the three outcomes (condomless sex, transactional sex, and frequent alcohol use). Separate multivariable logistic regression models were conducted to assess the relationship between emotional IPV and the three outcomes of interest, adjusting for age, current educational status, household assets, lifetime physical IPV, and HPTN 068 intervention arm. We also assessed the association between the sub-domains of emotional IPV (verbal abuse and threats) with the three outcomes of interest, adjusting for the aforementioned covariates.

Institutional Review Board approval was obtained from the University of North Carolina at Chapel Hill, the University of the Witwatersrand Human Research Ethics Committee and the University of California, San Francisco. All studies were conducted in accordance with the principles outlined in the Declaration of Helsinki. Participants who reported IPV or other abuse during the study were offered support through local public-sector social workers.

## Results

The mean age of participants at the post-trial HPTN 068 visit was 20 years, and 88.7% were currently in school or had graduated high school (Table 1). One quarter (25.1%) reported ever experiencing emotional IPV (threats and/or verbal IPV) in their lifetime, with verbal IPV (22.0%) being more common than threats from a partner (10.9%). Nearly one-quarter of participants (21.3%) reported condomless sex in the past 3 months, while 12.6% reported transactional sex since the last study visit, and 11.4% reported frequent alcohol use. Compared to participants who did not report lifetime experiences of emotional IPV, participants with lifetime experiences of emotional IPV were more likely to be older (20.1 years vs. 20.3 years; t-test p = 0.02), report lifetime physical IPV (43.3% vs. 56.7%, Pearson’s χ^2^ p ≤ 0.001), and were less likely to be currently in school or have graduated high school (89.8% vs. 85.2%, Pearson’s χ^2^ p = 0.005) (Table 1). Compared to those who did not report lifetime emotional IPV, AGYW who reported lifetime emotional IPV were also more likely to report condomless sex in the past 3 months (19.2% vs. 27.5%, Pearson’s χ^2^ p ≤ 0.001), transactional sex since the last study visit (9.6% vs. 21.4%, Pearson’s χ^2^ p ≤ 0.001), and frequent alcohol use (9.6% vs. 16.9%, Pearson’s χ^2^ p ≤ 0.001) (Table 1). Compared to those who did not report lifetime experiences of verbal IPV, those who did were more likely to report condomless sex (19.4% vs. 28.0%, Pearson’s χ^2^ p ≤ 0.001), transactional sex (10.3% vs. 20.6%, Pearson’s χ^2^ p ≤ 0.001) and frequent alcohol use (9.8% vs. 17.1%, Pearson’s χ^2^ p ≤ 0.001). Finally, compared to those who did not report lifetime experiences of threats from an intimate partner, those who did were also more likely to report condomless sex (20.6% vs.27.0%, Pearson’s χ^2^ p = 0.031), transactional sex (10.2% vs. 31.6%, Pearson’s χ^2^ p ≤ 0.001) and frequent alcohol use (10.9% vs. 15.7%, Pearson’s χ^2^ p = 0.037).

**Table 1:** Characteristics of AGYW participating in the HPTN 068 post-trial visit by lifetime experiences of emotional and economic intimate partner violence (IPV): Mpumalanga province, South Africa 2015–2017 (N = 1942)

Participant characteristics	Overall sample (n = 1942) n(%) or mean (SD)	No lifetime emotional violence (n = 1455) n(%) or mean (SD)	Lifetime emotional violence (n = 487) n(%) or mean (SD)	p values (χ^2^, or t-test)
Mean age at visit, (SD)	20.1 (1.4)	20.1 (1.4)	20.3 (1.5)	p = 0.02
Currently in school or graduated high school	1722 (88.7)	1307 (89.8)	415 (85.2)	p = 0.005
Mean household assets (out of 27 durable goods), (SD)	17.9 (7.1)	18.0 (7.2)	17.5 (6.9)	p = 0.22
HPTN intervention arm	1008 (51.9)	771 (52.9)	237 (48.7)	p = 0.09
Lifetime history of emotional intimate partner violence (IPV)	487 (25.1)	–	–	–
Lifetime history of verbal IPV	428 (22.0)	–	–	–
Lifetime history of threats from a partner	211 (10.9)	–	–	–
Lifetime physical IPV	937 (46.6)	630 (43.3)	276 (56.7)	p < 0.001
Condomless sex in the past 3 months	413 (21.3)	279 (19.2)	134 (27.5)	p < 0.001
Transactional sex since last visit	244 (12.6)	140 (9.6)	104 (21.4)	p < 0.001
Frequent alcohol use	220 (11.4)	138 (9.6)	82 (16.9)	p < 0.001

In adjusted analyses (Table 2), any emotional IPV was significantly associated with increased odds of condomless sex in the past 3 months (adjusted odds ratio (aOR): 1.47; 95% confidence interval (CI): 1.15, 1.87), transactional sex since the last visit (aOR: 2.32; 95% CI: 1.74, 3.08) and frequent alcohol use (aOR: 1.88; 95% CI: 1.39, 2.53). When considering subdomains of emotional violence, verbal IPV was significantly associated with increased odds of condomless sex (aOR: 1.48; 95% CI: 1.15, 1.89), transactional sex (aOR: 2.02; 95% CI: 1.51, 2.71) and frequent alcohol use (aOR: 1.87; 95% CI: 1.37, 2.55) in adjusted analyses, while threats was significantly associated with increased odds of transactional sex (aOR: 3.67; 95% CI: 2.62, 5.14).

**Table 2:** Adjusted associations between emotional intimate partner violence (IPV) and HIV risk behaviors (N = 1942)

	Condomless sex in past 3 months aOR^a^ (95% CI)	Transactional sex since last visit aOR^a^ (95% CI)	Frequent alcohol use aOR^a^ (95% CI)
Emotional intimate partner violence (IPV)	1.47 (1.15, 1.87)**	2.32 (1.74, 3.08)***	1.88 (1.39, 2.53)***
a. Verbal IPV	1.48 (1.15, 1.89)**	2.02 (1.51, 2.71)***	1.87 (1.37, 2.55)***
b. Threats	1.28 (0.91, 1.78)	3.67 (2.62, 5.14)***	1.48 (0.98, 2.23)

*p < 0.05; **p < 0.01; ***p < 0.001

^a^Adjusted for age at post-trial visit, current educational status, lifetime physical IPV, household assets, and HPTN 068 intervention arm

## Discussion

Among a cohort of AGYW in rural Mpumalanga province, South Africa, lifetime experiences of emotional IPV were common and significantly associated with increased odds of condomless sex, transactional sex, and frequent alcohol use—all risk factors for HIV. These results support and extend the small, but growing, body of research demonstrating that emotional IPV is associated with HIV risk independent of physical and sexual IPV, and thus merits additional attention in violence reduction programs [16, 22, 23]. To our knowledge, this is one of the first studies to demonstrate a positive association between emotional IPV and transactional sex and frequent alcohol use among AGYW in sub-Saharan Africa. Further, we found that the verbal abuse and threats sub-domains of emotional IPV were associated with different HIV risk behaviors, highlighting the potential importance of distinguishing between the various forms of emotional IPV in future research and programmatic efforts.

The relationship between emotional IPV and increased HIV risk behaviors could operate through a few paths. First, past research has demonstrated a relationship between emotional IPV and poor mental health outcomes including depression, anxiety, post-traumatic stress disorder and suicidality [24–26]. Poor mental health, in turn, has been associated with higher HIV incidence and increased HIV risk behaviors such as condomless sex, transactional sex, and alcohol use among women including AGYW [27–31]. A cross-sectional study among adult women from the United States (U.S.) found that PTSD symptomology mediated the relationship between emotional IPV and HIV risk behaviors [32]. Future research is needed to explore whether, and which, poor mental health outcomes mediate the relationship between emotional IPV and HIV risk behaviors among AGYW in sub-Saharan Africa.

Second, the relationship between emotional IPV and condomless sex, in particular, could operate through a path of reduced agency or self-efficacy to negotiate condom use as women who experience IPV may avoid asking their partner to use a condom for fear of being physically abused, sexually assaulted, or accused of infidelity [33]. Physical and sexual IPV have been associated with reduced condom negotiation self-efficacy among women [34]—an important determinant of condom use [35, 36]. Emotional IPV has also been linked to diminished self-esteem and self-efficacy among women [37], and reduced condom negotiation [38]. Evidence also points to the deleterious effects of gender inequitable norms at the community-level on condom negotiation self-efficacy among women. An analysis of pooled data from Demographic and Health Surveys from 22 countries in sub-Saharan Africa found that community-wide endorsement of gender norms condoning violence against women was negatively associated with condom use at last sex among women [39]. The authors argue that these inequitable gender norms lead to a prevailing sense of ‘everyday violence’ [40], that undermines women’s self-efficacy to negotiate condom use [39]. It is possible that the proximate context of inequitable gender norms in South Africa [41], and the associated sense of ‘everyday violence,’ explains the relationship between emotional IPV and condom use among AGYW in this setting. Future research is needed to explore this pathway.

Our finding that verbal IPV and threats were not associated with the same HIV risk behaviors provides additional nuance to our results that warrants further attention. We found that verbal IPV, defined as being insulted, belittled, or humiliated by a partner, was associated with all three HIV risk behaviors, while threats were only associated with transactional sex. Considering that the prevalence of threats was much lower than the prevalence of verbal IPV, it is possible that we did not have enough power to detect significant differences in the effects of threats on HIV risk behaviors. However, it is also possible that these findings indicate that verbal IPV and threats operate in distinct ways to influence HIV risk behaviors, suggesting the need to distinguish between the various forms of emotional IPV in future research and programmatic efforts. Future research is needed to explore the differential effects of verbal IPV and threats on HIV risk behaviors using larger sample sizes and longitudinal study designs.

Our findings related to the influence of verbal IPV on HIV risk behaviors build upon a scant number of studies from the U.S. which have found that verbal abuse is associated with heavy alcohol consumption and reduced condom use among women [26, 38]. Again, it is possible that these relationships work through paths of poor mental health outcomes and reduced condom negotiation self-efficacy, but future research is needed to explore this among this population. To our knowledge, this is one of the first studies to demonstrate that verbal abuse and threats are associated with transactional sex. Verbal abuse and threats from a partner may contribute to psychological trauma and poor mental health outcomes, which have been associated with engaging in transactional sex among adolescent girls in sub-Saharan Africa [27]. Past research suggests that women who turn to substance use as a coping mechanism to deal with the trauma of violence [42], may use transactional sex to sustain this behavior [43]. South African women have also described how experiences of abuse changes the way they view relationships, limiting their ability to trust men, and increasing their perception that a woman should gain something tangible from their relationships [43]. It is also important to note that we were unable to establish temporal order, due to the cross-sectional design of this study. It is therefore possible that AGYW who engage in transactional sex may be more likely to experience verbal abuse and threats from their partners. Longitudinal research is needed to further explore the impact of verbal IPV and threats on HIV risk behaviors among AGYW in South Africa. Future studies should also employ qualitative methods to understand AGYW’s lived experience of verbal IPV and threats and how such experiences may shape HIV-related risk behaviors.

The results from this study have several implications for future research and programmatic efforts designed to address IPV and prevent HIV among AGYW. First, our findings suggest that it is important for HIV and sexual and reproductive health research and programs to focus on understanding emotional IPV and its deleterious effects on HIV risk, independent from physical and sexual IPV. Second, there is an urgent need for longitudinal research to assess this relationship over time, and potential causal mechanisms, which can inform intervention approaches. Third, research is needed to further explore our finding that verbal IPV and threats were associated with different HIV risk behaviors. Such studies should use larger sample sizes and longitudinal study designs to tease apart the differential effects of the emotional IPV subdomains on HIV risk behaviors. Fourth, very few HIV prevention interventions for AGYW in sub-Saharan Africa have focused on addressing IPV, and those that do have primarily targeted physical and/or sexual IPV [44]. This study emphasizes the critical importance of adequately assessing and addressing experiences of emotional IPV in HIV prevention interventions and programmatic efforts among AGYW.

## Limitations

Findings are based on cross-sectional associations, which limits causal interpretation of the relationship between emotional IPV and HIV risk behaviors. We were also unable to adjust for lifetime experiences of sexual IPV, which has also been associated with HIV risk [13], as it was not assessed at all follow-up visits during the trial. Thus, future research must consider how lifetime experiences of sexual IPV may confound the association between emotional IPV and HIV risk behaviors. Additionally, our measures of emotional IPV did not allow us to assess when the violence occurred during a young woman’s life. Future research should explore whether recent experiences of emotional IPV have different effects on HIV risk behaviors compared to cumulative lifetime experiences among this population. Because our measures of emotional IPV and HIV risk behaviors were self-report, it is possible that AGYW underreported such experiences and behaviors due to social desirability bias. Past research has documented underreporting of IPV and sexual risk behavior in similar settings [45, 46]. This may have resulted in the underestimation of the prevalence of lifetime emotional IPV and HIV risk behaviors among this population and limited our power to detect significant differences particularly in the threats sub-domain of emotional IPV. We sought to mitigate this potential bias by using the ACASI survey tool, which has been found to make participants more comfortable in disclosing sensitive information [47]. Finally, this study was conducted among young, cis-gender women from rural South Africa so findings may not be generalizable to adult women, transgender women, or other racial/ethnic groups in other settings.

## Conclusion

This study found that emotional IPV was associated with increased odds of condomless sex, transactional sex, and frequent alcohol use among AGYW participating in the HPTN 068 cohort in rural South Africa. We also found evidence of different relationships between emotional IPV sub-domains and HIV risk behaviors, signaling the potential importance of distinguishing the various forms of emotional IPV in future research. Longitudinal research is needed to assess the impact of emotional IPV, and its sub-domains, on HIV risk behaviors over-time, and to examine causal pathways. Increased focus on emotional IPV in HIV prevention interventions and programmatic efforts for AGYW in sub-Saharan Africa is critical to improve their health and well-being.

## Data Availability

The HPTN 068 data we analyzed in this paper contains sensitive health information, including HIV status and sexual behavior data, of young women in rural South Africa. In accordance with the protocol of this secondary data analysis reviewed and approved by the University of California, San Francisco Institutional Review Board, we cannot make the dataset publicly available to third party users. However, data access is managed by FHI360 and data requests can be made by contacting Erica Hamilton at EHamilton@fhi360.org.

## References

[1] UNAIDS (2018). Miles to go: closing gaps, breaking barriers, righting injustices.

[2] Decker MR, Latimore AD, Yasutake S, Haviland M, Ahmed S, Blum RW (2015). Gender-based violence against adolescent and young adult women in low- and middle-income countries. J Adolesc Health.

[3] Centers for Disease Control and Prevention. Preventing intimate partner violence 2017 Available from: https://www.cdc.gov/violenceprevention/pdf/ipv-factsheet.pdf.

[4] Birdthistle I, Tanton C, Tomita A, de Graaf K, Schaffnit SB, Tanser F (2019). Recent levels and trends in HIV incidence rates among adolescent girls and young women in ten high-prevalence African countries: a systematic review and meta-analysis. Lancet Glob Health.

[5] National Department of Health (NDoH) SSASS, South African Medical Research, Council (SAMRC) aI (2019). South Africa Demographic and Health Survey 2016.

[6] Selin A, DeLong SM, Julien A, MacPhail C, Twine R, Hughes JP (2019). Prevalence and associations, by age group, of IPV among AGYW in rural South Africa. Sage Open.

[7] Jewkes RK, Dunkle K, Nduna M, Shai N (2010). Intimate partner violence, relationship power inequity, and incidence of HIV infection in young women in South Africa: a cohort study. Lancet (London, England).

[8] Durevall D, Lindskog A (2014). Intimate partner violence and HIV in ten sub-Saharan African countries: what do the Demographic and Health Surveys tell us?. Lancet Global Health..

[9] Li Y, Marshall CM, Rees HC, Nunez A, Ezeanolue EE, Ehiri JE (2014). Intimate partner violence and HIV infection among women: a systematic review and meta-analysis. J Int AIDS Soc.

[10] Kouyoumdjian FG, Calzavara LM, Bondy SJ, O’Campo P, Serwadda D, Nalugoda F (2013). Intimate partner violence is associated with incident HIV infection in women in Uganda. AIDS (London, England).

[11] DeLong SM. Intimate partner violence and incident HIV among adolescent girls and young women in Agincourt area, South Africa [Dissertation]. In press 2018.

[12] UNAIDS (2019). Women and HIV: a spotlight on adolescent girls and young women.

[13] Dunkle KL, Decker MR (2013). Gender-based violence and HIV: reviewing the evidence for links and causal pathways in the general population and high-risk groups. Am J Reprod Immunol (New York, NY: 1989).

[14] Kouyoumdjian FG, Findlay N, Schwandt M, Calzavara LM (2013). A systematic review of the relationships between intimate partner violence and HIV/AIDS. PLoS One..

[15] Jewkes R (2010). Emotional abuse: a neglected dimension of partner violence. Lancet.

[16] Gibbs A, Dunkle K, Willan S, Jama-Shai N, Washington L, Jewkes R (2019). Are women’s experiences of emotional and economic intimate partner violence associated with HIV-risk behaviour? A cross-sectional analysis of young women in informal settlements in South Africa. AIDS Care.

[17] Dude AM (2011). Spousal intimate partner violence is associated with HIV and other STIs among married Rwandan women. AIDS Behav.

[18] Breiding MJ, Basile KC, Smith SG, Black MC, Mahendra RR (2015). Intimate partner violence surveillance: uniform definitions and recommended data elements, version 2.0.

[19] Pettifor A, MacPhail C, Selin A, Gomez-Olive FX, Rosenberg M, Wagner RG (2016). HPTN 068: a randomized control trial of a conditional cash transfer to reduce HIV infection in young women in South Africa-study design and baseline results. AIDS Behav.

[20] Kahn K, Collinson MA, Gomez-Olive FX, Mokoena O, Twine R, Mee P (2012). Profile: agincourt health and socio-demographic surveillance system. Int J Epidemiol.

[21] World Health Organization (2005). WHO multi-country study on women’s health and domestic violence against women: summary report of initial results on prevalence, health outcomes and women’s responses.

[22] Wingood GM, DiClemente RJ (1997). The effects of an abusive primary partner on the condom use and sexual negotiation practices of African-American women. Am J Public Health.

[23] Ali B, Mittal M, Schroder A, Ishman N, Quinton S, Boekeloo B (2020). Psychological violence and sexual risk behavior among predominantly African American women. J Interpers Violence.

[24] Gibbs A, Dunkle K, Jewkes R (2018). Emotional and economic intimate partner violence as key drivers of depression and suicidal ideation: a cross-sectional study among young women in informal settlements in South Africa. PLoS One.

[25] Pico-Alfonso MA, Garcia-Linares MI, Celda-Navarro N, Blasco-Ros C, Echeburua E, Martinez M (2006). The impact of physical, psychological, and sexual intimate male partner violence on women’s mental health: depressive symptoms, posttraumatic stress disorder, state anxiety, and suicide. J Womens Health (Larchmt).

[26] Coker AL, Davis KE, Arias I, Desai S, Sanderson M, Brandt HM (2002). Physical and mental health effects of intimate partner violence for men and women. Am J Prev Med.

[27] Larsen A, Kinuthia J, Lagat H, Sila J, Abuna F, Kohler P (2020). Depression and HIV risk behaviors among adolescent girls and young women seeking family planning services in Western Kenya. Int J STD AIDS.

[28] Jackson JM, Seth P, DiClemente RJ, Lin A (2015). Association of depressive symptoms and substance use with risky sexual behavior and sexually transmitted infections among African American female adolescents seeking sexual health care. Am J Public Health.

[29] Cavanaugh CE, Hansen NB, Sullivan TP (2010). HIV sexual risk behavior among low-income women experiencing intimate partner violence: the role of posttraumatic stress disorder. AIDS Behav.

[30] Fidalgo TM, da Silveira ED, da Silveira DX (2008). Psychiatric comorbidity related to alcohol use among adolescents. Am J Drug Alcohol Abuse.

[31] Goin DE, Pearson RM, Craske MG, Stein A, Pettifor A, Lippman SA (2020). Depression and incident HIV in adolescent girls and young women in HIV prevention trials network 068: targets for prevention and mediating factors. Am J Epidemiol.

[32] Overstreet NM, Willie TC, Hellmuth JC, Sullivan TP (2015). Psychological intimate partner violence and sexual risk behavior: examining the role of distinct posttraumatic stress disorder symptoms in the partner violence-sexual risk link. Womens Health Issues.

[33] Fox AM, Jackson SS, Hansen NB, Gasa N, Crewe M, Sikkema KJ (2007). In their own voices: a qualitative study of women’s risk for intimate partner violence and HIV in South Africa. Violence Against Women.

[34] Swan H, O’Connell DJ (2012). The impact of intimate partner violence on women’s condom negotiation efficacy. J Interpers Violence.

[35] Baele J, Dusseldorp E, Maes S (2001). Condom use self-efficacy: effect on intended and actual condom use in adolescents. J Adolesc Health.

[36] Giles M, Liddell C, Bydawell M (2005). Condom use in African adolescents: the role of individual and group factors. AIDS Care.

[37] Matheson FI, Daoud N, Hamilton-Wright S, Borenstein H, Pedersen C, O’Campo P (2015). Where did she go? The transformation of self-esteem, self-identity, and mental well-being among women who have experienced intimate partner violence. Womens Health Issues.

[38] Peasant C, Sullivan TP, Ritchwood TD, Parra GR, Weiss NH, Meyer JP (2018). Words can hurt: the effects of physical and psychological partner violence on condom negotiation and condom use among young women. Women Health.

[39] Tsai AC, Subramanian SV (2012). Proximate context of gender-unequal norms and women’s HIV risk in sub-Saharan Africa. AIDS.

[40] Shannon K, Kerr T, Allinott S, Chettiar J, Shoveller J, Tyndall MW (2008). Social and structural violence and power relations in mitigating HIV risk of drug-using women in survival sex work. Soc Sci Med.

[41] Pulerwitz J, Gottert A, Kahn K, Haberland N, Julien A, Selin A (2019). Gender norms and HIV testing/treatment uptake: evidence from a large population-based sample in South Africa. AIDS Behav.

[42] Kaysen D, Dillworth TM, Simpson T, Waldrop A, Larimer ME, Resick PA (2007). Domestic violence and alcohol use: trauma-related symptoms and motives for drinking. Addict Behav.

[43] Dunkle KL, Jewkes RK, Brown HC, Gray GE, McIntryre JA, Harlow SD (2004). Transactional sex among women in Soweto, South Africa: prevalence, risk factors and association with HIV infection. Social Sci Med (1982).

[44] Hosek S, Pettifor A (2019). HIV prevention interventions for adolescents. Curr HIV/AIDS Rep.

[45] Palermo T, Bleck J, Peterman A (2014). Tip of the iceberg: reporting and gender-based violence in developing countries. Am J Epidemiol.

[46] Gallo MF, Behets FM, Steiner MJ, Thomsen SC, Ombidi W, Luchters S (2007). Validity of self-reported ‘safe sex’ among female sex workers in Mombasa, Kenya–PSA analysis. Int J STD AIDS.

[47] Mensch BS, Hewett PC, Jones HE, Luppi CG, Lippman SA, Pinho AA (2008). Consistency in women’s reports of sensitive behavior in an interview mode experiment, Sao Paulo, Brazil. Int Fam Plan Perspect.

